# Shisa3 brakes resistance to EGFR-TKIs in lung adenocarcinoma by suppressing cancer stem cell properties

**DOI:** 10.1186/s13046-019-1486-3

**Published:** 2019-12-04

**Authors:** Jiahui Si, Yuanyuan Ma, Ji Wang Bi, Ying Xiong, Chao Lv, Shaolei Li, Nan Wu, Yue Yang

**Affiliations:** Department of Thoracic Surgery II, Key Laboratory of Carcinogenesis and Translational Research (Ministry of Education), Peking University Cancer Hospital and Institute, 52 Fucheng Road, Haidian District, Beijing, 100142 People’s Republic of China

**Keywords:** Shisa3, Cancer stem cells, EGFR-TKI resistance, FGFR, mTOR

## Abstract

**Background:**

Although EGFR tyrosine kinase inhibitors (EGFR-TKIs) are beneficial to lung adenocarcinoma patients with sensitive EGFR mutations, resistance to these inhibitors induces a cancer stem cell (CSC) phenotype. Here, we clarify the function and molecular mechanism of shisa3 as a suppressor that can reverse EGFR-TKI resistance and inhibit CSC properties.

**Methods:**

The suppresser genes involved in EGFR-TKI resistance were identified and validated by transcriptome sequencing, quantitative real-time PCR (qRT-PCR) and immunohistochemistry. Biological function analyses, cell half maximal inhibitory concentration **(**IC50), self-renewal, and migration and invasion capacities, were detected by CCK8, sphere formation and Transwell assays. Tumorigenesis and therapeutic effects were investigated in nonobese diabetic/severe combined immunodeficiency (nod-scid) mice. The underlying mechanisms were explored by Western blot and immunoprecipitation analyses.

**Results:**

We found that low expression of shisa3 was related to EGFR-TKI resistance in lung adenocarcinoma patients. Ectopic overexpression of shisa3 inhibited CSC properties and the cell cycle in the lung adenocarcinoma cells resistant to gefitinib/osimertinib. In contrast, suppression of shisa3 promoted CSC phenotypes and the cell cycle in the cells sensitive to EGFR-TKIs. For TKI-resistant PC9/ER tumors in nod-scid mice, overexpressed shisa3 had a significant inhibitory effect. In addition, we verified that shisa3 inhibited EGFR-TKI resistance by interacting with FGFR1/3 to regulate AKT/mTOR signaling. Furthermore, combinational administration of inhibitors of FGFR/AKT/mTOR and cell cycle signaling could overcome EGFR-TKI resistance associated with shisa3-mediated CSC capacities in vivo.

**Conclusion:**

Taken together, shisa3 was identified as a brake to EGFR-TKI resistance and CSC characteristics, probably through the FGFR/AKT/mTOR and cell cycle pathways, indicating that shisa3 and concomitant inhibition of its regulated signaling may be a promising therapeutic strategy for reversing EGFR-TKI resistance.

## Introduction

EGFR tyrosine kinase inhibitors (EGFR-TKIs) have been an effective therapy for lung adenocarcinoma patients with activating mutations; however, therapeutic resistance to EGFR-TKIs inevitably develops [1]. Of note, cancer stem cells (CSCs), which can regrow after clinical management, play an important role in resistance to chemotherapy, targeted therapy and immunotherapy [2, 3]. Lung cancer CSCs can be identified by the surface markers CD133, CD44, ALDH1 and ABCG2 and are regulated by Notch, Wnt and cell cycle signaling pathways, which are critical for maintaining drug resistance [3]. Anti-CSC therapeutics targeting surface markers and associated pathways in different cancer types have been developed in animal models and investigated in clinical trials [4, 5]. Thus, a comprehensive understanding of the molecular mechanism of CSC regulation in EGFR-TKI resistance might provide a novel strategy for treatment intervention.

The role of tumor suppressor genes in modulating the EGFR-TKI response has attracted the attention of researchers. FOXO3a, as a suppressor, has been shown to increase EGFR-TKI sensitivity and reduce CSC properties [6]. Shisa, as a tumor suppressor, has been discovered to antagonize the CSC-associated Wnt pathway and FGF signaling by interacting with immature forms of their receptors [7]. Sox2, an essential transcriptional factor of CSCs, was upregulated after FGFR1 activation [8], and FGFR1 signaling has been shown to contribute to the maintenance of CSC properties by interacting with the Hippo/YAP1 pathway in lung cancer [9]. Shisa3 has been reported to accelerate the degradation of β-catenin, a key component of Wnt signaling, which inhibits tumorigenesis, invasion and metastasis in lung cancer [10].

In addition to the acquisition of EGFR mutations, such as T790 M (resistance to gefitinib, erlotinib and ecotinib) [11] and C797S (resistance to osimertinib) [12], the activation of receptor tyrosine kinases (RTKs) combined with triggering downstream signaling pathways, including RAS and mitogen-activated protein kinase (MAPK), phosphoinositide 3-kinase (PI3K) and Akt, or signal transducer and activator of transcription 3 (STAT3), has been recognized to drive EGFR-TKI resistance in lung cancer [13]. Recently, it has been reported that the use of Akt inhibitor plus EGFR-TKIs led to suppressed growth in lung adenocarcinoma models of TKI resistance [14]. In the presence of mTOR activation, TKI-resistant lung adenocarcinoma cells showed a better response to combinational treatment with mTOR and TKIs [15].

In the current study, we screened shisa3 as a tumor suppressor with decreased expression in lung adenocarcinoma patients with EGFR-TKI resistance. Intriguingly, shisa3 combined with gefitinib/osimertinib dramatically inhibited tumor growth of TKI-resistant lung adenocarcinomas. Shisa3 blockade drove EGFR-TKI resistance, cell cycle arrest and CSC enrichment by regulating the FGFR-dependent Akt/mTOR signaling pathway. Further studies indicated that TKIs combined with cell cycle and mTOR inhibitors significantly restrained tumor growth in TKI-resistant lung adenocarcinoma xenografts with upregulated shisa3 and downregulated Ki67. Taken together, targeting shisa3-regulated networks may provide a novel treatment strategy for reversing TKI drug resistance and CSC activities.

## Methods

### Specimens

In this study, all the samples were obtained from the lung adenocarcinoma patients who received surgery and neoadjuvant treatment before resection at Peking University Cancer Hospital (Beijing, China). All participants provided written informed consent, and the studies were approved by the Ethics Committee of Peking University Cancer Hospital. Paraffin tumor tissues from 45 cases of EGFR mutant stage III lung adenocarcinoma patients who had received EGFR-TKI treatment (gefitinib or icotinib) after surgery were investigated; these patients were enrolled from December 2009 to January 2013. Response to treatment was evaluated according to the Response Evaluation Criteria in Solid Tumors.

In all, 102 paraffin tumor tissue samples with EGFR mutation were collected from these lung adenocarcinoma patients who underwent surgery from January 2009 to November 2011. In addition, 38 pairs of frozen tumor tissues and normal tissues from the lung adenocarcinoma patients with EGFR mutation were involved in 2012. All patients were regularly followed, and the clinical outcomes of all the patients were obtained.

### Transcriptome sequencing (RNA-seq)

Total mRNA was extracted using TRIzol reagent (#15596018, Invitrogen, Carlsbad, CA, USA) according to the manufacturer’s instructions. RNA-seq and subsequent data analyses were performed by the Beijing Institute of Genome Research, Chinese Academy of Sciences (Beijing, China). Briefly, 2 μg RNA per sample was used to construct a cDNA library and was sequenced on the Illumina HiSeq 2500 with 125–150 bp paired-end reads following the manufacturer’s recommendations. Bowtie software was used to align the raw reads to the *Homo sapiens* genome sequences (NCBI). The false discovery rate (FDR, i.e., a probability of wrongly accepting a difference) of each gene was determined according to the Bonferroni correction method. Differential expression analysis was performed using the edgeR R package (2.6.2). An adjusted *P*-value < 0.05 and FDRs < 0.01 were set as the threshold for significantly differential expression.

### Quantitative real-time PCR (qRT-PCR)

Total RNA was isolated from tissues and cells using TRIzol reagent (Invitrogen) following the manufacturer’s instructions, and synthesis of cDNA from total RNA (2 μg) was performed using a commercially available kit (EasyScript First-Strand cDNA Synthesis SuperMix, Transgen Biotech, Beijing, China). qRT-PCR was performed with the LightCycler 480 SYBR Green I Master using a LightCycler 480 Real-Time PCR System (Roche, Mannheim, Germany). The relative expression level of genes was normalized to GAPDH. The fold change was calculated according to 2^-∆Ct^, in which ∆Ct = Ct _target_ - Ct _control_. All PCR assays were carried out in triplicate, and the mean of triplicates is reported. Primers are listed in Additional file 1: Table S1.

### Immunohistochemistry (IHC)

Formalin-fixed and paraffin-embedded lung adenocarcinoma samples were incubated with primary anti-shisa3 (1:1000 dilution; Thermo Fisher Scientific, Rockford, IL, USA), anti-FGFR1 (1:200 dilution; Abcam, Cambridge, MA) or Ki-67 (1:200 dilution; ZSGB-BIO, Beijing, China)overnight at 4 °C followed by IgG/HRP polymer (ZSGB-BIO) and diaminobenzidine substrate (ZSGB-BIO) in compliance with protocols. Staining results were independently evaluated by two experienced pathologists who were blinded to all clinical data.

### Cell lines and cell culture

The human lung adenocarcinoma cell lines PC9, HCC827 and H1975 were maintained in our laboratory. Cell lines were cultured in RPMI-1640 medium (Gibco BRL, Gaithersburg, MD) supplemented with 10% fetal bovine serum (Gibco BRL), 100 U/mL penicillin, and 100 g/mL streptomycin (Invitrogen, Grand Island, NY, USA) and incubated in a humidified incubator (37 °C) with 5% CO_2_. All cell lines were certified by short-tandem repeat (STR) analysis.

### Reagents and antibodies

Gefitinib (ZD1839), osimertinib (AZD9291), BGJ398, dactolisib (BEZ235) and PD0332991 were purchased from Selleck Chemicals (Houston, TX, USA). Reagents were formulated and stored according to the manufacturer’s protocols. The following primary antibodies were used: AKT (#9272), p-AKT S473 (#9271), mTOR (#2983), p-mTOR S2448 (#2971), S6K (#9205S), p-S6K Thr389 (#9202S), S6 (#2217), p-S6 Ser235/236 (#4858), and FGFR3 (#4574) from Cell Signaling Technology (Danvers, MA, USA); Shisa3 (#167069, for Western blot), FGFR1 (#ab10646) and p-FGFR3 Y724 (#ab155960) from Abcam (Cambridge, MA); p-FGFR1 T653/T654 (#06–1433) from Merck Millipore (Darmstadt, Germany), cyclin D1 (#sc-8396), CDK4 (#sc-23,896), CDK6 (#sc-7961), and p16 (#sc-166,760) from Santa Cruz Biotechnology (Santa Cruz, CA); β-actin (#HRP-60008) from Proteintech Group (Rosemont, IL, USA). The secondary HRP-conjugated goat anti-rabbit (#CW0103S) and anti-mouse antibodies (#CW0102S) were from CWBIO (Beijing, China).

### Generation of EGFR-TKI-resistant cells (PC9/ER)

PC9/ER cells were developed from the parental PC9 cells by 6 months of exposure to gefitinib, starting at 10 nM and increasing stepwise to 10 μM. At 80–90% confluence, the cells were detached with trypsin/EDTA (Gibco BRL) and divided into 2 parts. One part of the cells was frozen, and the other was reseeded into a new dish at doses 30–50% higher than the original. Control cells were treated parallel with vehicle (DMSO). The PC9/ER cells were validated to be resistant to gefitinib and osimertinib as shown in the results.

### Vector construction and transfection

The lentiviral vector of shisa3 was constructed by inserting a shisa3 cDNA (NM_001080505.2) fragment into a lentiviral shuttle vector. Shisa3 knockdown was accomplished using specific shRNAs targeting shisa3. The shRNA sequences were as follows: shRNA-shisa3#1: 5′-GTGGCTATTTATTGTTGCA-3′; shRNA-shisa3#2: 5′-GCTCCATCTTCATTGCGTT-3′; and shControl: 5′-TTCTCCGAACGTGTCAGGT-3′. The packing and purification of the lentiviral vectors were performed by the GenePharma Company (Shanghai, China). The indicated cells infected with the lentiviral vectors were selected with puromycin for 2 weeks.

Tet-on expression plasmids were constructed by pELNS-M2rtTA-IRES-Neo and plenti6-TREpitt-GFP vectors for shisa3 expression.

pENTER-shisa3-flag was constructed by cloning the coding sequence (CDS) of shisa3 into the pENTER plasmid using the restriction sites Asis I and Mlu I. For further experiments, PC9/ER cells were transiently transfected with this plasmid using Lipofectamine™ 3000 (Invitrogen) according to the manufacturer’s protocols.

### Western blot analysis

The proteins of cells were extracted using RIPA buffer containing a complete protease inhibitor cocktail (Roche, Mannheim, Germany). Protein concentrations were measured with a bicinchoninic acid (BCA) protein assay kit (Beyotime). Equal amounts of protein were separated with 8% or 10% SDS-PAGE and transferred to polyvinylidene fluoride (PVDF) membranes. After blocking with 5% BSA (Amresco) or fat-free milk, the membranes were probed with primary antibodies at 4 °C overnight followed by secondary antibodies at room temperature for 1 h. The proteins were then detected by chemiluminescence using Immobilon Western Chemiluminescent HRP Substrate (#WBKLS0500, Millipore) and visualized using Amersham Imager 600 (GE Healthcare, Chicago, IL).

### Microarray and computational analysis

PC9-shControl and PC9-shShisa3 cells were submitted to BoHao Bio-tech (Shanghai, China) for mRNA microarray analysis. Total RNA was purified with an RNeasy mini kit (Cat.# 74,106, QIAGEN, GmBH, Germany) and hybridized using the Gene Expression Hybridization Kit (Cat.# 5188–5242, Agilent Technologies, Santa Clara, CA, US). Data were extracted with Feature Extraction 10.7 software (Agilent Technologies). Genes that were up- or downregulated with > 2-fold change (FC) and a significant difference of *P* < .05 were further subjected to computational simulation by Ingenuity Pathway Analysis (IPA; QIAGEN, Valencia, CA, USA) online tools for an enrichment analysis.

### Cell viability

A total of 3000–5000 cells were seeded per well in 96-well plates and allowed to attach for 24 h. Following treatment, cell viability was measured using a CCK-8 commercial kit (#CK04, Dojindo, Japan) according to the manufacturer’s protocol, and the absorbance at 450 nm was measured using a spectrophotometer. The cell half maximal inhibitory concentration **(**IC50) was calculated using GraphPad software.

### Sphere formation assay

The cells were trypsinized and washed in phosphate-buffered saline (PBS), and single cells were plated in ultralow-attachment 96-well plates (Corning Inc., Life Sciences). DMEM/F-12 (Invitrogen) serum-free medium including 20 ng/mL basic fibroblast growth factor (Peprotech, Rocky Hill, NJ, USA), 20 ng/mL epidermal growth factor (Peprotech), B27 (Invitrogen), 10 ng/mL hepatocyte growth factor (Peprotech), and 1% methylcellulose (Sigma, MO, USA) was used to cultivate the cells for 12 days. The spheres were counted under a light microscope. Images are shown as representatives of three independent experiments. Sphere formation efficiency was calculated as follows: sphere formation efficiency = sphere/input cells × 100%.

### Migration and invasion assays

A total of 1.0 × 10^5^ cells per well in serum-free RPMI were placed in the upper chamber (Cat NO. 3422, Corning Costar, Cambridge, MA) with/without precoated Matrigel (Cat NO. 356234, BD Biosciences, San Jose, California, USA) following the manufacturer’s instructions. The lower chambers were filled with culture medium supplemented with 10% FBS. The indicated cells were allowed to migrate or invade through pores for 12 to 24 h at 37 °C. The total numbers of migrated or invaded cells in the lower chambers were fixed in paraformaldehyde (4%) and stained with 0.1% crystal violet for 5 min at room temperature and counted under a microscope.

### Cell cycle assay

The effect of shisa3 on cell cycle distribution was analyzed by flow cytometry. After being starved overnight and stimulated with complete medium for 24 h, cells were collected, washed with PBS, and fixed in 75% immediately precooled ethanol overnight at − 20 °C. After washing with PBS, cells were stained with propidium iodide (PI)/RNase (BD Biosciences) at room temperature for 15 min in the dark and analyzed by flow cytometry within 1 h. Quantitative cell cycle analysis was assessed with ModFit version 3.0 software (Verity Software House, Topsham, ME).

### Xenografts and treatments

Nonobese diabetic/severe combined immunodeficiency (nod-scid) mice were cared for in accordance with guidelines approved by the Ethics Committee of Animal Experiments of Peking University Cancer Hospital. A total of 3 × 10^6^ cells were subcutaneously injected into the right flank of 6-week-old female nod-scid mice (Beijing HFK Bioscience Co., Ltd., China). When tumors reached a size of approximately 100 mm^3^, the mice were randomized into several groups separately via oral gavage with different treatments. Tumors were measured every 4 days using calipers, and tumor volume was calculated using the formula volume = (length × width^2^)/2. At the end of the treatments, the mice were sacrificed with CO_2_, and the tumors were stripped for successive assays.

### Immunoprecipitation

Cells were washed twice in ice-cold PBS, harvested and lysed with RIPA lysis buffer (#P0013D, Beyotime, Wuhan, China) for immunoprecipitation experiments. For each sample, 1.5 mg of protein was incubated with Protein G Sepharose 4 Fast Flow (#17061802, GE Healthcare, UK) and anti-Flag antibody (#66008–2-Ig, Proteintech) or anti-IgG antibody (#A7028, Beyotime). After an overnight incubation at 4 °C, the beads were washed, and the final pellet was suspended in RIPA buffer. Bound proteins were eluted from the beads by heating and centrifugation and then analyzed by Western blot.

### Statistical analysis

The relationship between the expression of shisa3 and patients’ clinical variables was assessed using the χ ^2^-test and Fisher’s exact test. The disease-free survival (DFS) and overall survival (OS) of patients were estimated using the Kaplan-Meier method and the log-rank test. The Cox hazard proportional model was applied to multivariate analysis. All statistical analyses were performed using SPSS version 20.0 software (SPSS Inc., Chicago, IL), and images were plotted using GraphPad Prism 7 (GraphPad Software, La Jolla, CA, USA). Differences were analyzed using unpaired two-tailed t tests. All data are representative of 3 independent experiments and are illustrated as the means ± SDs. (*P* < 0.05 was considered statistically significant.)

## Results

### Decreased shisa3 is associated with EGFR-TKI resistance in lung adenocarcinoma

**Fig. 1:**
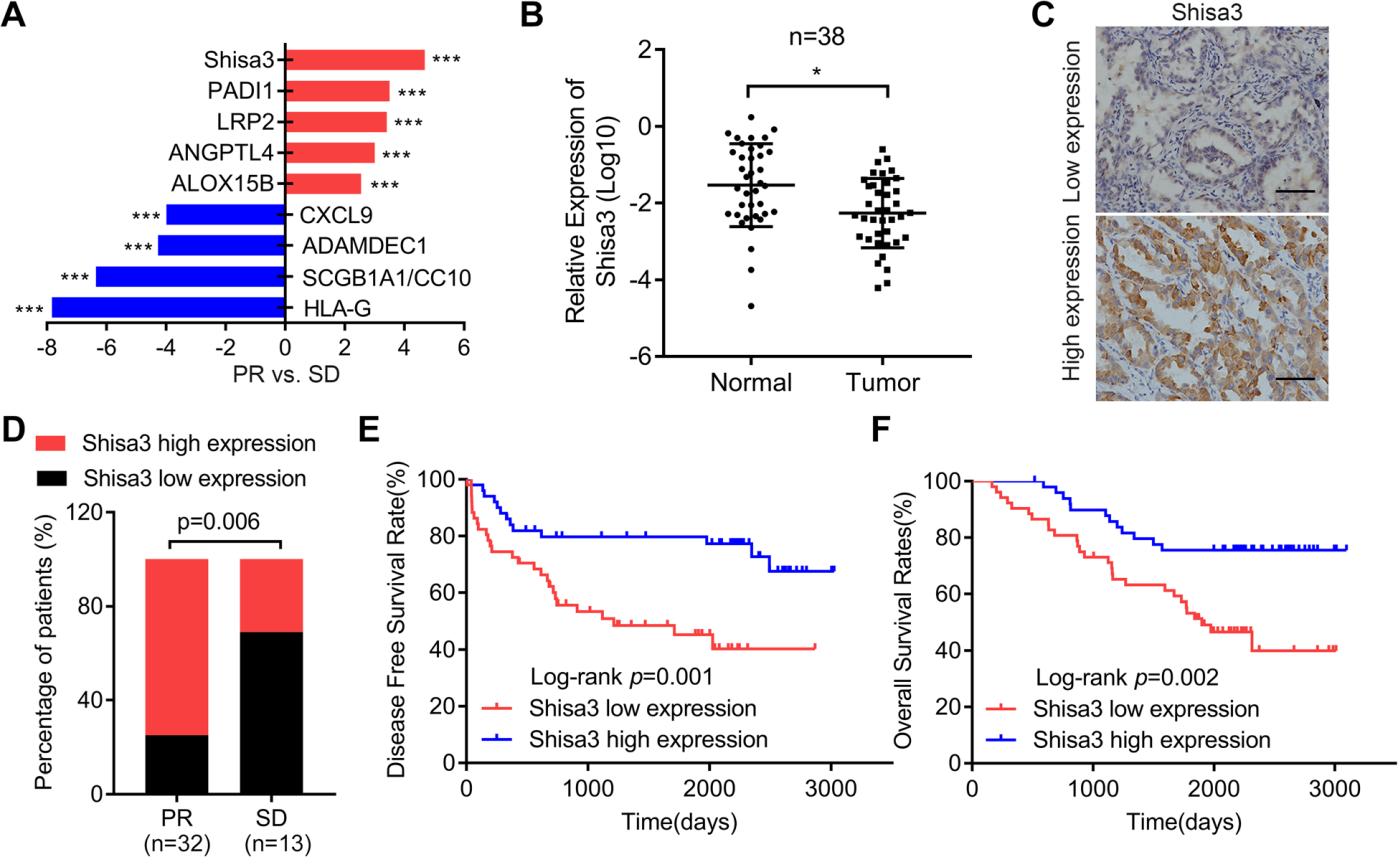
Shisa3 expression is associated with EGFR-TKI efficacy and prognosis in lung adenocarcinoma. **a**. The fold change of gene expression as indicated in 3 tumor tissues with PR compared to 3 tumor tissues with SD, and all the samples were derived from lung adenocarcinoma patients who received EGFR-TKI treatment by the method of transcriptome sequencing analysis. **b**. The relative expression of shisa3 in 38 paired normal tissues and tumor tissues of lung adenocarcinoma by qRT-PCR analysis. **c**. Representative images (× 200 magnification) of shisa3 staining by immunohistochemical analysis in tumor tissues. Scale bars, 50 μm. **d**. The percentages of samples with high and low expression of shisa3 in lung adenocarcinoma patients who received EGFR-TKI treatment (SD, *n* = 13; PR, *n* = 32) by immunohistochemical analysis. **e**, **f** Kaplan-Meier analysis of the DFS (**e**) and OS (**f**) of lung adenocarcinoma patients (*n* = 102) in connection with shisa3 expression. *p < 0.05; **p < 0.001; ***p < 0.0001

We used RNA-seq to screen gene expression levels in local advanced lung adenocarcinoma patients who received EGFR-TKI (gefitinib or ecotinib) therapy (Additional file 1; Table S2). There were 3 drug-sensitive patients with partial response (PR) and 3 drug-resistant patients with stable disease (SD). Herein, we found that 5 genes (Shisa3, PADI1, LRP2, ANGPTL4 and ALOX15B) were upregulated and 4 genes (CXCL9, ADAMDEC1, SCGB1A1/CC10, HLA-G) were downregulated in PR tumor tissues compared to SD tumor tissues (Fig. 1a). Owing to the genes that could inhibit EGFR-TKI resistance, we focused on shisa3, which was the most upregulated gene in PR samples with a 4.68-fold increase. In addition, shisa3 was validated to be highly expressed in normal tissues compared to paired tumor tissues of lung adenocarcinoma with EGFR activating mutations (Fig. 1b, *n* = 38).

We further determined by IHC detection whether shisa3 was associated with the therapeutic effect of EGFR-TKIs in lung adenocarcinoma patients with EGFR mutations (*n* = 45) who received gefitinib/ecotinib treatment. Based on the divided groups of low shisa3 expression (−/+) and high shisa3 expression (++/+++) (Fig. 1c), an increased rate of high expression was observed in EGFR-TKI-sensitive patients (PR: 75%) compared to EGFR-TKI-resistant patients (SD: 31%) (Fig. 1d). Thus, we demonstrated that patients with high expression of shisa3 have a better response to EGFR-TKIs, indicating that shisa3 may be used to predict the efficacy of TKI therapy in lung adenocarcinoma patients.

A previous study of 69 samples from non-small-cell lung cancer (NSCLC) patients revealed that shisa3 was positively correlated with better prognosis [10]. Subsequently, we investigated shisa3 status in 102 tissue samples from lung adenocarcinoma patients with EGFR mutations. There was no significant difference between shisa3 expression level and sex, age, smoking history, venous invasion, and differentiation (Table 1). We found that low expression of shisa3 was related to later TNM stage and lymph node metastasis. We further obtained evidence that low expression of shisa3 was associated with shorter DFS and OS (Fig. 1e-f). Shisa3 was identified as an independent OS factor for lung adenocarcinoma by univariate and multivariate Cox regression analyses (Table 2).

**Table 1:** Clinicopathological variables and shisa3 expression in lung adenocarcinoma patients (*n* = 102)

Variable	Case no. (%)	Shisa3		*P* value
		Low expression	High expression	
Gender				0.712
Male	43 (42.2%)	21 (48.8%)	22 (51.2%)	
Female	59 (57.8%)	31 (52.5%)	28 (47.5%)	
Age (years)				0.436
≤60	47 (46.1%)	22 (46.8%)	25 (53.2%)	
> 60	55 (53.9%)	30 (54.5%)	25 (45.5%)	
Smoking history				0.874
Yes	62 (60.8%)	32 (51.6%)	30 (48.4%)	
No	40 (39.2%)	20 (50.0%)	20 (50.0%)	
Venous invasion				0.390
Negative	80 (78.4%)	39 (48.8%)	41 (51.2%)	
Positive	22 (21.6%)	13 (59.1%)	9 (40.9%)	
Differentiation				0.789
Poor	36 (35.3%)	19 (52.8%)	17 (47.2%)	
Moderate/well	66 (64.7%)	33 (50.0%)	33 (50.0%)	
TNM stage				0.098
I/II	74 (72.5%)	34 (45.9%)	40 (54.1%)	
III	28 (27.5%)	18 (64.3%)	10 (35.7%)	
N stage				**0.013**
0	63 (61.8%)	26 (41.3%)	37 (58.7%)	
1/2	39 (38.2%)	26 (66.7%)	13 (33.3%)	

Bold values are significant (*p*<0.05)

**Table 2:** Univariate and multivariate cox regression analyses for overall survival (OS) in lung adenocarcinoma patients (*n* = 102)

Variables	Univariate analysis		Multivariate analysis	
	HR (95%CI)	*P* value	HR (95%CI)	*P* value
Gender	0.593(0.319–1.103)	0.099	NA	
Age	1.036(0.553–1.914)	0.911	NA	
Smoking history	1.522(0.818–2.831)	0.185	NA	
Venous invasion	1.848(0.939–3.636)	0.075	NA	
Differentiation	0.572(0.307–1.068)	0.079	NA	
TNM stage	2.649(1.413–4.968)	**0.002**	3.452(1.009–11.814)	**0.048**
N stage	1.946(1.046–3.621)	**0.035**	NA	
Shisa3	2.802(1.421–5.527)	**0.003**	2.606(1.298–5.235)	**0.007**

*HR* hazard ratio, *CI* confidence interval, bold values are significant (*p*<0.05)

These data suggested that shisa3 may drive sensitivity to EGFR-TKIs in EGFR-mutant lung adenocarcinoma.

### The established EGFR-TKI-resistant cells induced the CSC phenotype

**Fig. 2:**
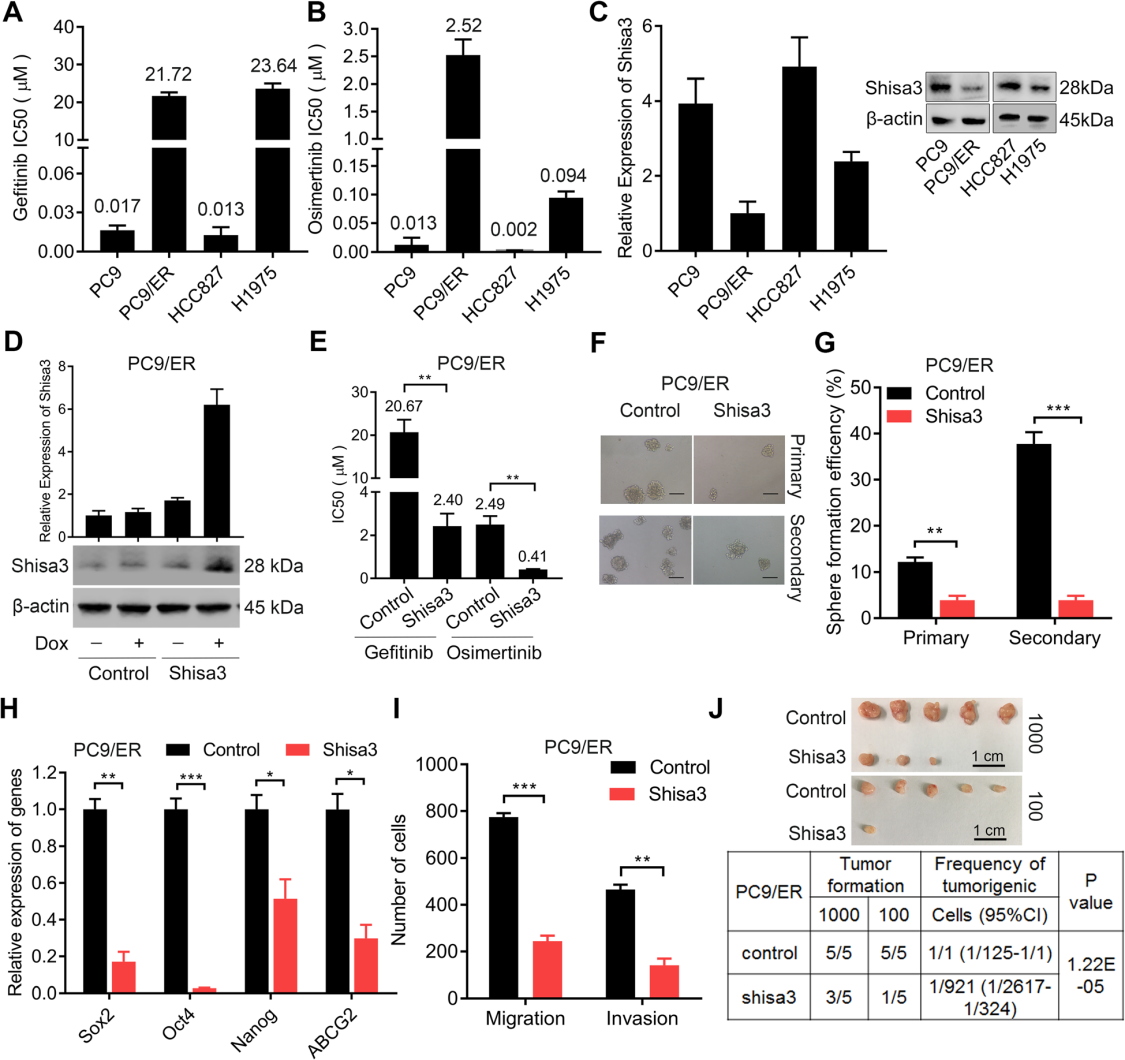
Shisa3 decreases EGFR-TKI resistance and inhibits a CSC phenotype. **a**, **b**. The histograms show the IC50 of PC9, PC9/ER, HCC827 and H1975 cells for gefitinib (**a**) and osimertinib (**b**). **c**. Shisa3 transcription levels and protein expression were analyzed by qRT-PCR (left panel) and Western blot (right panel) in PC9, PC9/ER, HCC827 and H1975 cells. β-actin was used as a loading control. **d**. The mRNA and protein levels of shisa3 were measured in PC9/ER cells transfected with shisa3 in Tet-on inducible vector (2 μg/ml of doxycycline-induction) by qRT-PCR and western blot. **e**. The histogram shows the IC50 for gefitinib and osimertinib in PC9/ER cells expressing shisa3 induced by doxycycline (2 mg/ml) treatment for 48 h. **f**. Representative the primary and secondary sphere images of PC9/ER cells. Scale bars, 100 μm. **g**. The histogram demonstrates the primary and secondary sphere formation efficiencies in PC9/ER and PC9/ER cells overexpressing shisa3. **h**. Lower expression levels of CSC-related markers were observed by qRT-PCR in shisa3-overexpressing PC9/ER cells than in control cells. **i**. The graph demonstrates the number of migrated and invasive PC9/ER and shisa3-overexpressing PC9/ER cells. **j**. Representative tumorigenic images formed by transplanting 1 × 10^2^ and 1 × 10^3^ PC9/ER or PC9/ER-shisa3 cells into nod-scid mice (upper panel). Tumorigenic frequency was calculated by extreme limiting dilution analysis (ELDA). **e**, **g**, **h** and **i**: *p < 0.05; **p < 0.001; ***p < 0.0001

Consistent with previous studies [16–18], we verified that PC9 (gefitinib IC50 = 0.017 ± 0.003 μM, osimertinib IC50 = 0.013 ± 0.012 μM) and HCC827 (gefitinib IC50 = 0.013 ± 0.006 μM, osimertinib IC50 = 0.002 ± 0.001 μM) cells were sensitive to EGFR-TKIs and that H1975 (gefitinib IC50 = 23.64 ± 1.42 μM, osimertinib IC50 = 0.094 ± 0.011 μM) cells were resistant to a first-generation EGFR-TKI (gefitinib) but sensitive to a third-generation EGFR-TKI (osimertinib) (Fig. 2a-b). Next, we generated EGFR-TKI-resistant PC9/ER cells derived from PC9 cells, showing a 1315.6-fold increase in IC50 for gefitinib and a 196.3-fold increase in IC50 for osimertinib. In addition, compared with HCC827 cells, PC9/ER cells demonstrated a 1698.8-fold increase in gefitinib IC50; compared with HCC827 cells, PC9/ER cells exhibited a 1429.0-fold increase in osimertinib IC50. Among the EGFR hotspot analyses, only a sensitive deletion mutation of Exon 19 was identified in PC9/ER cells (Additional file 1; Table S3). In view of the decreased expression of shisa3 in lung adenocarcinoma tissues that were resistant to EGFR-TKI treatment, we detected this gene expression in lung adenocarcinoma cells with variable IC50 to gefitinib/osimertinib. Lower expression of shisa3 was detected in PC9/ER cells compared to PC9, HCC827 and H1975 cells (Fig. 2c).

Considering that tumor cells with EGFR-TKI resistance may manifest stem-cell-like properties [19, 20], we then analyzed whether PC9/ER cells exhibited a CSC phenotype. The primary and secondary sphere formation efficiencies were elevated in PC9/ER cells compared with PC9 cells (Additional file 1: Figure S1A-B). The expression levels of CSC-related factors, including Sox2, Oct4, Nanog and ABCG2, were increased in PC9/ER cells (Additional file 1: Figure S1C). Migration and invasion properties were enhanced in PC9/ER cells compared to PC9 cells (Additional file 1: Figure S1D-E). We then performed a limited dilution assay by transplanting PC9 and PC9/ER cells into nod-scid mice. PC9/ER cells, which showed increased tumorigenic frequency, formed more and larger tumors than PC9 cells (Additional file 1: Figure S1F). Based on the reported mechanisms in EGFR-TKIs resistance [21], we then investigated Met, HER-2, PTEN and EMT related signaling in PC9/ER and the parental PC9 cells. Among those factors, Met expression level and EMT activity (decrease of E-cadherin, and increase of N-cadherin and vimentin) were enhanced in the PC9/ER cells (Additional file 1: Figure S1G). In addition, the CSC related makers including CD133, CD44 and ALDH1A1 were up-regulated in the PC9/ER cells, compared to the parental PC9 cells (Additional file 1: Figure S1H).

These results suggested that PC9/ER cells with decreased expression of shisa3 demonstrated dramatic resistance to EGFR-TKIs and showed an enhanced CSC phenotype.

### Overexpressed shisa3 attenuates EGFR-TKI resistance and suppresses the CSC phenotype

To investigate the biological effect of shisa3, we overexpressed and validated the increased expression of this protein in PC9/ER and H1975 cells (Additional file 1: Figure S2A). Shisa3 resulted in a decreased proliferation in PC9/ER cells (Additional file 1: Figure S2B) and H1975 cells (Additional file 1: Figure S2C). Since shisa3 as a tumor suppressor gene, shisa3 was established in the Tet-on inducible system to transfect the PC9/ER and H1975 cells. The expression of shisa3 was significantly up-regulated after doxycycline induction for 48 h (Fig. 2d, and Additional file 1: Figure S2D). Shisa3 led to a decreased IC50 for gefitinib and osimertinib in PC9/ER cells (Fig. 2e). Moreover, inhibitory effect of gefitinib was enhanced in the H1975 cells overexpressing shisa3 induced by doxycycline (Additional file 1: Figure S2E).

We then examined whether shisa3 might be a suppressor that depresses the CSC phenotype. We observed a significant decrease in primary and secondary sphere formation efficiencies in PC9/ER cells overexpressing shisa3 (Fig. 2f-g). Lower expression levels of Sox2, Oct4, Nanog and ABCG2 were observed in shisa3-overexpressing PC9/ER cells than in PC9/ER-control cells (Fig. 2h). CD133, CD44 and ALDH1A1 were down-regulated in the shisa3-overexpression PC9/ER cells (Additional file 1: Figure S2F). When shisa3 was overexpressed in PC9/ER cells, fewer cells exhibited migration and invasion capacities (Additional file 1: Figure S2G, Fig. 2i). We then performed a limited dilution assay in nod-scid mice, showing fewer and smaller tumors and lower tumorigenic frequencies in shisa3-overexpressing PC9/ER cells (Fig. 2j).

### Knockdown of shisa3 triggers EGFR-TKI resistance and enhances the CSC phenotype

**Fig. 3:**
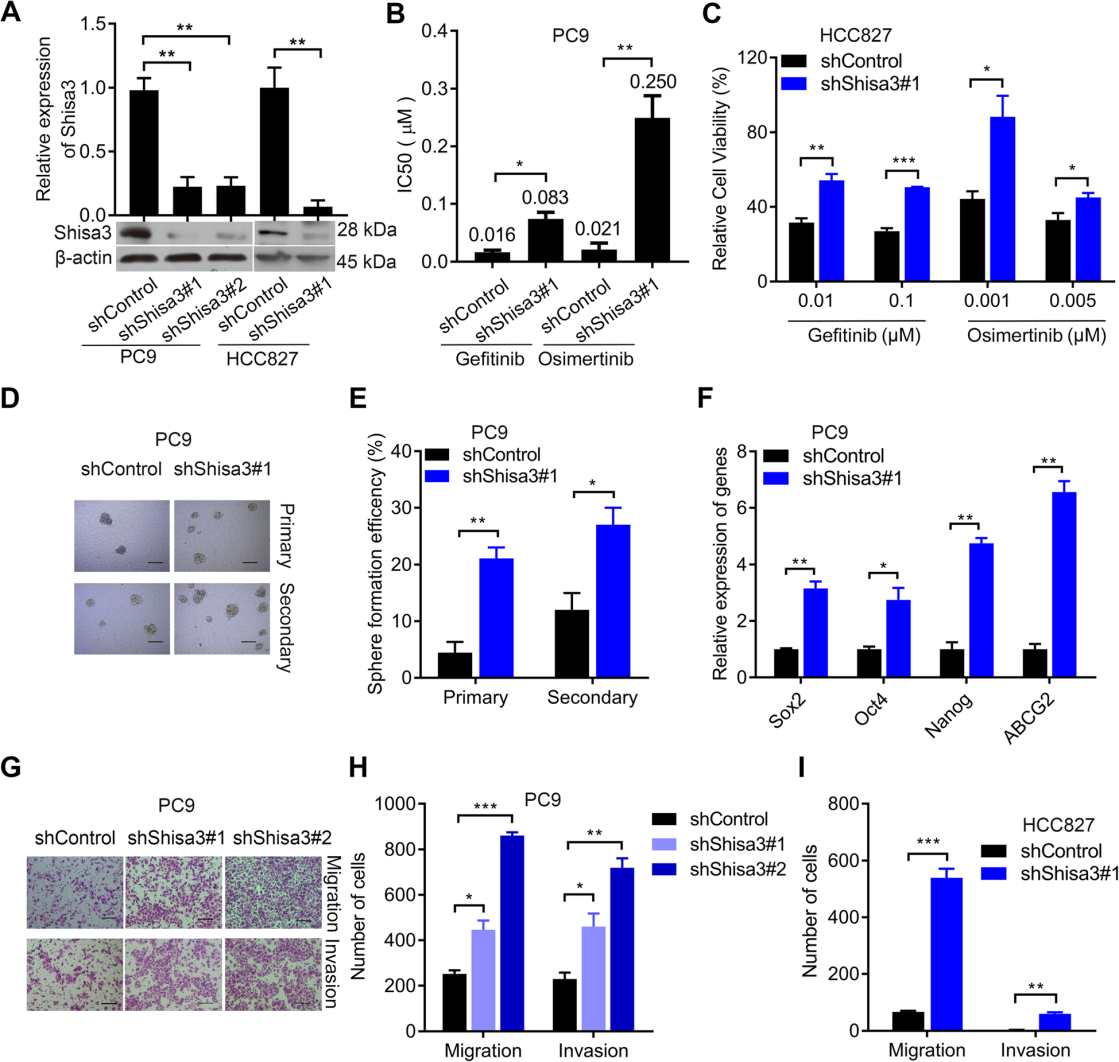
Suppression of shisa3 induces EGFR-TKI resistance and promotes a CSC phenotype. **a**. QRT-PCR and Western blot analysis of shisa3 expression in PC9 and HCC827 cells stably infected with shRNAs containing PC9-shControl or shisa3-targeting sequences (PC9-ShShisa3#1 and #2). **b**. IC50 values for gefitinib and osimertinib are shown in the bar graph in PC9-ShControl and PC9-ShShisa3#1 cells. **c**. HCC827 cells with or without shisa3 inhibition were treated with gefitinib (0.01 μM, 0.1 μM) or osimertinib (0.001 μM, 0.005 μM), and relative cell viability was determined by CCK-8 assay. **d**. Representative the primary and secondary sphere images in the transfected PC9 cells. Scale bars, 100 μm. **e**. The histogram shows the primary and secondary sphere formation efficiencies in PC9-shControl and PC9-shShisa3#1 cells. **f**. Higher expression levels of CSC-related markers were observed by qRT-PCR in shisa3-downregulated PC9 cells than in control cells. **g**. Transwell assays demonstrated the number of migrated and invasive PC9 cells transfected with the indicated shRNA. Scale bars, 100 μm. **h**, **i**. The graphs show the number of migrated and invasive PC9 (**h**) and HCC827 (**i**) cells transfected with the indicated shRNA, respectively. **a**, **b**, **c**, **e**, **f**, **h** and **i**: *p < 0.05; **p < 0.001; ***p < 0.0001

Next, we downregulated shisa3 in PC9 and HCC827 cells by transfecting these cells with lentiviral-based shRNAs. The shisa3 expression level was validated to be suppressed by Shshisa3#1 and Shshisa3#2 (Fig. 3a). Decreased shisa3 led to a 4.48-fold increase in gefitinib IC50 and an 11.75-fold increase in osimertinib IC50 in PC9 cells (Fig. 3b). Knockdown of shisa3 increased cell viabilities in HCC827 cells treated with gefitinib and osimertinib (Fig. 3c).

Then, we analyzed how CSC properties changed in PC9 and HCC827 cells after shisa3 knockdown. It was shown that decreased expression of shisa3 enhanced the primary and secondary sphere formation efficiencies (Fig. 3d-e). Sox2, Oct4, Nanog and ABCG2 expression levels were higher in the PC9 cells transfected with Shshisa3#1 than in the cells transfected with shControl (Fig. 3f). CD133, CD44 and ALDH1A1 were also induced in the PC9 cells with knock-down of shisa3 (Additional file 1: Figure S2H). The results showed more migratory and invasive cells in PC9 and HCC827 cells with suppressed shisa3 (Fig. 3g-i, Additional file 1: Figure S2I).

The above data indicated that shisa3 functions to reverse EGFR-TKI resistance and attenuate CSC potential.

### Shisa3 interacts with FGFR to impact EGFR-TKI sensitivity

**Fig. 4:**
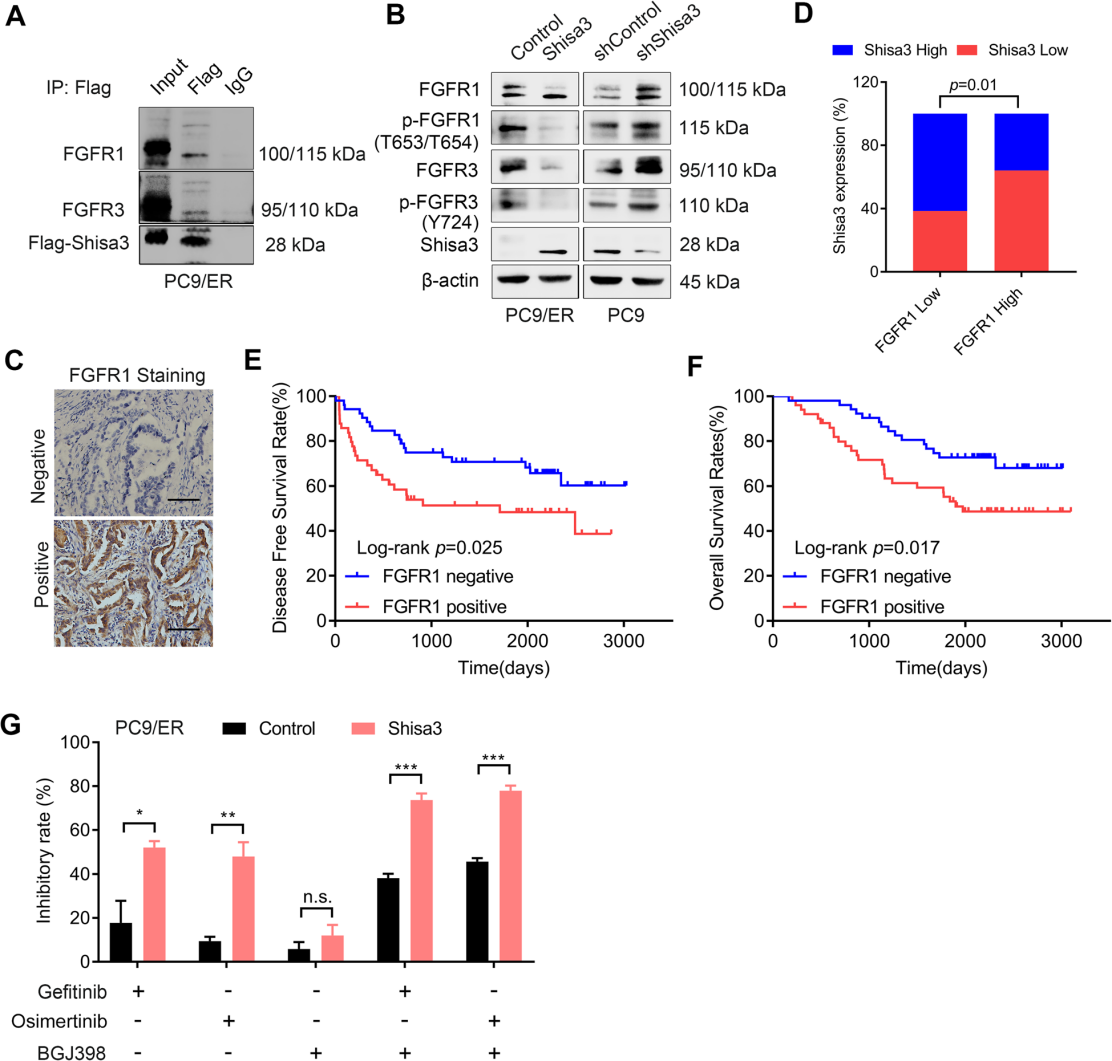
Shisa3 interacts with FGFR to impact EGFR-TKI sensitivity. **a**. Cell lysates from PC9/ER cells transfected with Flag-Shisa3 were immunoprecipitated with Flag or IgG and blotted with FGFR1, FGFR3, and Flag antibodies. **b**. Western blot analysis in cellular extracts of indicated cells. **c**. Representative images (× 200 magnification) of FGFR1 staining by immunohistochemical analysis in tumor tissues. Scale bars, 50 μm. **d**. Histogram of the negative relationship between shisa3 and FGFR1 expression in lung adenocarcinoma tissues with EGFR mutations (*n* = 102). **e**, **f**. Kaplan-Meier analysis of the DFS (**e**) and OS (**f**) of lung adenocarcinoma patients (n = 102) in connection with FGFR1 expression. **G**. PC9/ER-control and PC9/ER-shisa3 cells were incubated with the following agents: gefitinib (0.5 μM), osimertinib (0.1 μM), BGJ398 (1 μM), or a combination of gefitinib/osimertinib and BGJ398. **g**: n.s. *p* > 0.05; *p < 0.05; **p < 0.001; ***p < 0.0001

Shisa has been reported to physically interact with FGFR and inhibit its protein maturation [7]; therefore, we explored whether shisa3 interacted with FGFR to modulate the EGFR-TKI response. We further verified the bands of FGFR1 and FGFR3 in immunoprecipitation by shisa3-Flag in PC9/ER cells (Fig. 4a). We observed that overexpression of shisa3 dramatically reduced FGFR1, FGFR3, phosphorylated (p) FGFR1 (p-FGFR1) and p-FGFR3 expression levels in PC9/ER cells, and suppression of this gene induced these two receptors and their phosphorylation in PC9 cells (Fig. 4b). IHC was performed to identify FGFR1 expression in lung adenocarcinoma (Fig. 4c). A negative relationship was shown in the lung adenocarcinoma tissues with EGFR mutations (*n* = 102, Fig. 4d). In those patients, higher expression of FGFR1 was associated with shorter DFS and OS (*n* = 102, Fig. 4e-f). Then, the FGFR1/3 inhibitor-BGJ398 was used to treat the PC9/ER cells. In presence of shisa3, BGJ398 increased response to gefitinib and osimertinib by 21.63 and 25.87% in the PC9/ER cells, respectively (Fig. 4g).

These results indicated that shisa3 interacted with FGFR1/3 and inhibit their activation to increase the EGFR-TKI response to a certain extent.

### Shisa3 controls EGFR-TKI resistance by inhibiting FGFR/AKT/mTOR and cell cycle signaling

**Fig. 5:**
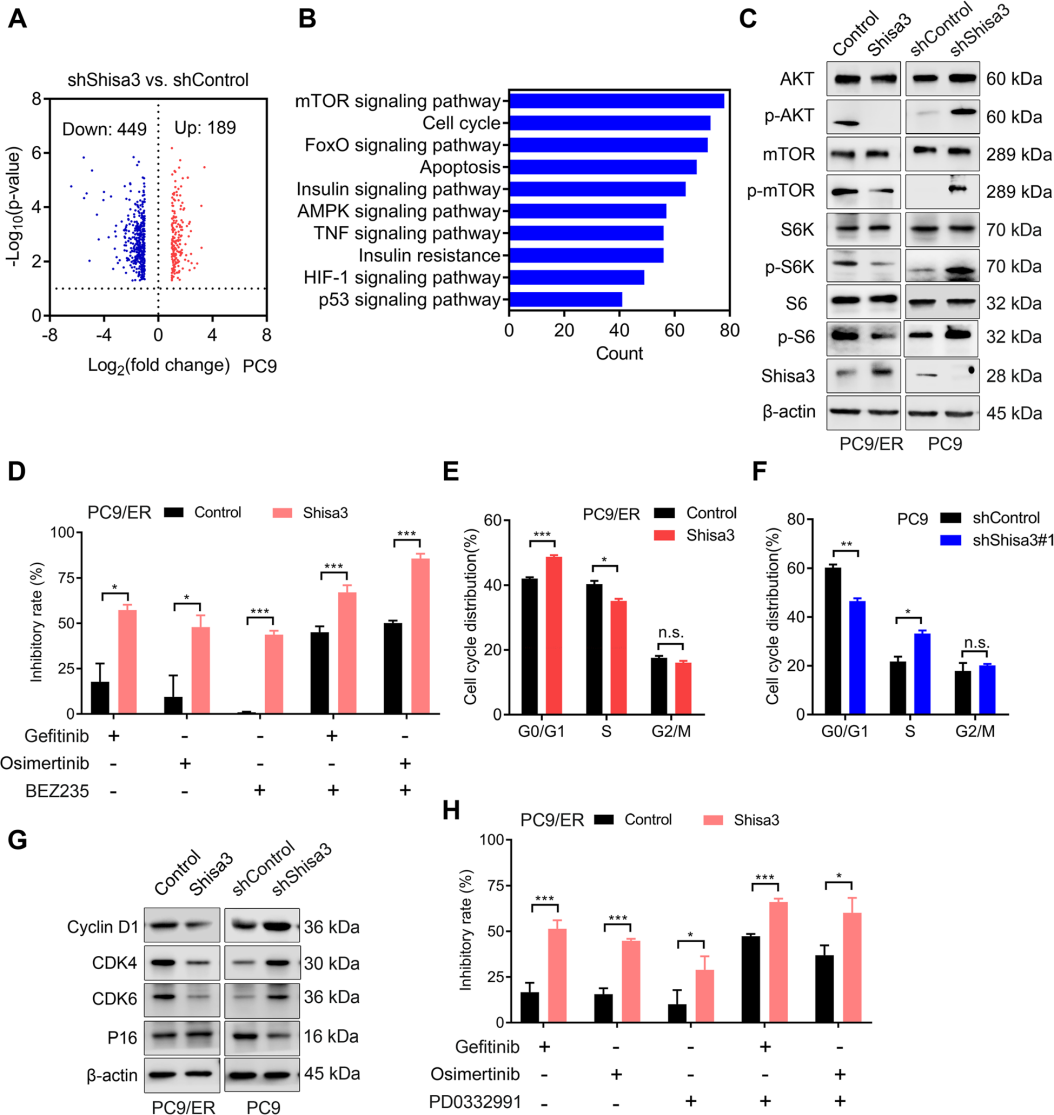
Shisa3 controls EGFR-TKI resistance via inhibition of FGFR/AKT/mTOR and cell cycle signaling. **a**. Volcano plot depicting a log transformation plot of the fold difference (x-axis) and the *P*-value (y-axis) of indicated genes between PC9-shShisa3 and PC9- shControl cells. **b**. Signaling pathways were enriched in the PC9-shShisa3 cells over the control cells by Kyoto Encyclopedia of Genes and Genomes (KEGG) analysis using a microarray. **c**. Western blots show AKT, mTOR, S6K and S6 and their phosphorylation signals in PC9/ER cells and PC9 cells with the indicated shisa3 expression. **d**. PC9/ER-control and PC9/ER-shisa3 cells were incubated with the following agents: gefitinib (0.5 μM), osimertinib (0.1 μM), BEZ235 (0.01 μM), or a combination of gefitinib/osimertinib and BEZ235. **e**. Flow-cytometric analysis of cell cycle distribution in PC9/ER-control and PC9/ER-shisa3 cells. **f**. The percentage of the cell population at G0/G1, S, and G2/M phases in PC9-shControl and PC9-shShisa3 cells. **g**. Effect of shisa3 expression on cell cycle-related proteins. **h**. PC9/ER-control and PC9/ER-shisa3 cells were incubated with the following agents: gefitinib (0.5 μM), osimertinib (0.1 μM), PD0332991 (12 μM), or a combination of gefitinib/osimertinib and PD0332991. **d**, **e**, **f** and **h**: n.s. *p* > 0.05; *p < 0.05; ***p < 0.0001

To dissect the molecular mechanism of the shisa3-mediated EGFR-TKI response, we performed microarray of RNA analysis in knocked-down shisa3 PC9 cells with 449 upregulated genes and 189 downregulated genes (Fig. 5a). Signaling pathways, including mTOR, cell cycle, FoxO, apoptosis, insulin and AMPK, were enriched when shisa3 was suppressed (Fig. 5b).

The increased genes associated with mTOR signaling were listed in the Additional file 1; Table S4. Furthermore, overexpression of shisa3 resulted in significant inhibition of p-AKT, p-mTOR, p-S6K and p-S6 in PC9/ER cells (Fig. 5c, left panel). In contrast, the loss of shisa3 increased the activation of p-AKT, p-mTOR, p-S6K and p-S6 in PC9 cells (Fig. 5c, right panel). Then, we investigated whether a mTOR signaling inhibitor (BEZ235) could influence EGFR-TKI sensitivity in lung adenocarcinoma cells. Gefitinib, osimertinib, BEZ235 or a combination of gefitinib or osimertinib and BEZ235 suppressed PC9/ER cells by 17.82 ± 10.06%, 9.49 ± 11.84%, 0.97 ± 0.36%, 45.15 ± 3.19% or 50.21 ± 1.32%, and this inhibitory effect could be enhanced to 57.41 ± 2.9%, 47.97 ± 6.48%, 43.75 ± 2.2%, 67.09 ± 3.92% or 85.56 ± 2.6% in PC9/ER cells overexpressing shisa3, respectively (Fig. 5d).

Based on the microarray data (Fig. 5b, Additional file 1; Table S5), we validated whether shisa3 regulates cell cycle distribution. Cell cycle arrest was observed in the shisa3-overexpressing PC9/ER cells with increased G0/G1 stage and decreased S and G2/M stages (Fig. 5e). In contrast, suppression of shisa3 promoted the cell cycle in PC9 cells (Fig. 5f). In addition, lower expression of cyclin D1, CDK4 and CDK6 was observed in the shisa3-overexpressing PC9/ER cells, and higher expression of these proteins was observed in PC9 cells with suppressed shisa3 (Fig. 5g). We then detected how the cell cycle inhibitor palbociclib (PD0332991) influenced EGFR-TKI sensitivity. PD0332991 increased the inhibitory rate of gefitinib/osimertinib in PC9/ER cells overexpressing shisa3 (Fig. 5h).

AKT activation has been reported to mediate FGFR inhibitor resistance [22]. These data suggested that the shisa3-mediated increased response to EGFR-TKIs might be associated with the inactivation of FGFR/AKT/mTOR and cell cycle signaling.

### Targeting shisa3-regulated signaling attenuated EGFR-TKI resistance

**Fig. 6:**
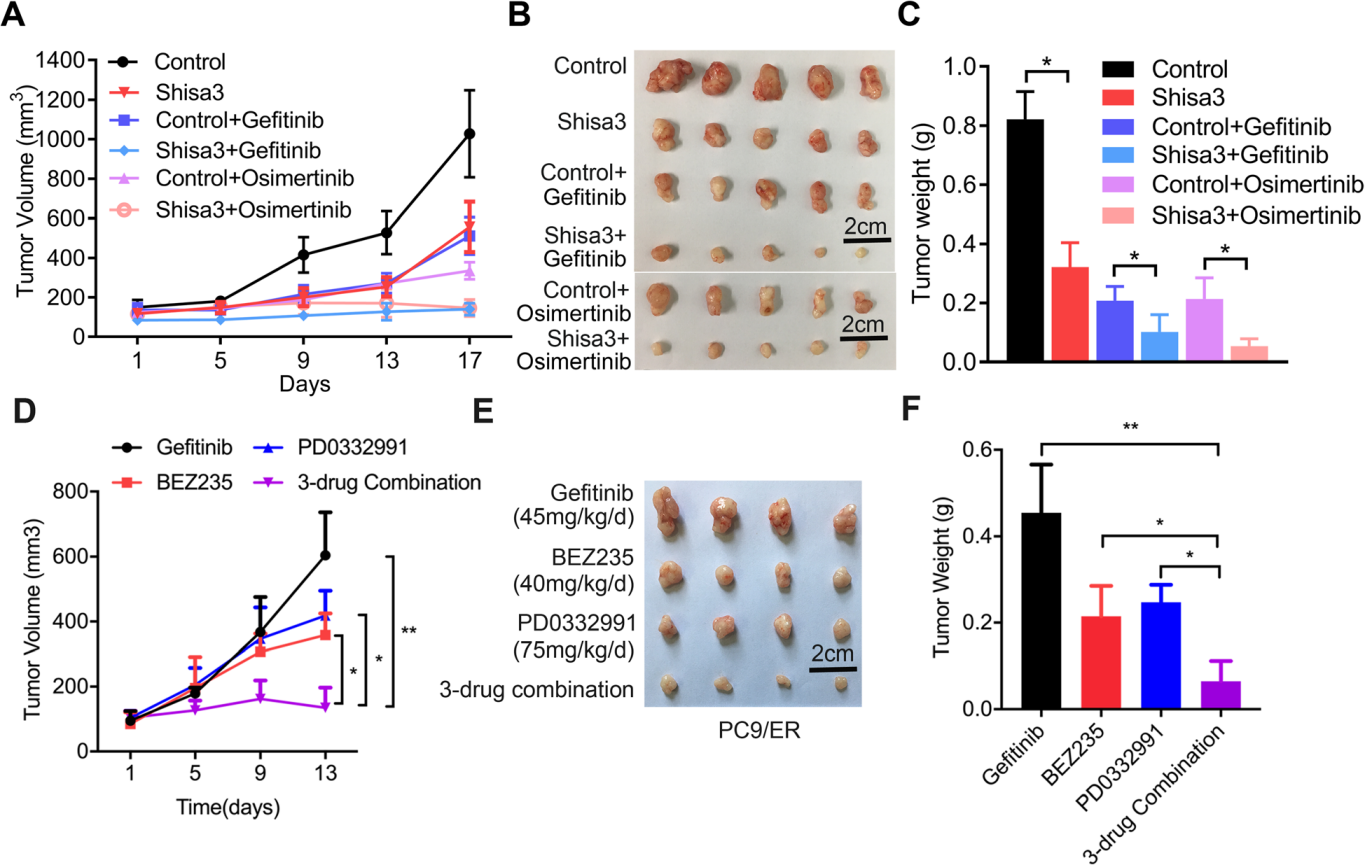
Targeting shisa3-regulated signaling restored EGFR-TKI sensitivity. **a**. Growth curves in the PC9/ER-control and PC9/ER-shisa3 models with control (1% Tween 80 in PBS), gefitinib (60 mg/kg/d) or osimertinib (25 mg/kg/d) treatments at the indicated time points. Data are presented as the means ± SDs; *n* = 5. **b**. Representative xenograft images of PC9/ER-control and PC9/ER-shisa3 tumors with or without gefitinib and osimertinib treatments. **c**. Histogram of tumor weights in PC9/ER-control and PC9/ER-shisa3 tumors with or without gefitinib and osimertinib treatment. **d**. Growth curves in the PC9/ER models with gefitinib (45 mg/kg/d), BEZ235 (40 mg/kg/d), PD0332991 (75 mg/kg/d), or a 3-drug combination treatment at the indicated time points. Data are presented as the means ± SDs; *n* = 4. **e**. Representative xenograft images of PC9/ER tumors with the indicated treatments. **f**. Histogram of tumor weights in PC9/ER tumors with the indicated treatments. **c**, **d** and **f**: *p < 0.05; **p < 0.001

Firstly, we tested inhibitory effect of gefitinib (15 mg/kg/day) and osimertinib (5 mg/kg/day) on the formed tumors of PC9 cells. As shown in the Additional file 1: Figure S3A-C, gefitinib or osimertinib could significantly control tumor growth of PC9 cells, with 89 and 99% inhibitory effect, respectively. To assess the role of shisa3 in inhibiting tumor growth of lung adenocarcinoma cells with EGFR-TKI resistance, we injected PC9/ER-control and PC9/ER-shisa3 cells into nod-scid mice. Until the tumor volume reached 100 mm^3^, the mice were subsequently treated with vehicle (1% Tween 80 in PBS) as a control group, gefitinib (60 mg/kg/d) or osimertinib (25 mg/kg/d) by oral gavage for 14 days. Compared to the control group, the groups treated with gefitinib, osimertinib or shisa3 overexpression alone showed inhibited tumor growth by 55.23, 67.20 and 57.91%, respectively, and the combination of gefitinib or osimertinib and shisa3 overexpression even dramatically suppressed tumor growth by 87.70 and 87.16%, respectively (Fig. 6a-b). Lower tumor weights were detected in the shisa3-overexpressing PC9/ER tumors or the PC9/ER tumors with EGFR-TKI (gefitinib/osimertinib) treatment, and even the lowest tumor weights were observed in the shisa3-overexpressing PC9/ER tumors with gefitinib or osimertinib treatment (Fig. 6c).

Based on the above data, we further studied the therapeutic effect via targeting shisa3-regulated signaling, and the control group was showed as Fig. 6a-c. Compared to gefitinib treatment, BEZ235 or PD0332991 could inhibit tumor growth, and the combination of gefitinib, BEZ235 and PD0332991 even dramatically suppressed tumor growth in the EGFR-TKI resistant xenografts of PC9/ER (Fig. 6d-f). These data indicated that shisa3-regulated signaling may be a brake for lung adenocarcinoma with EGFR-TKI resistance.

**Fig. 7:**
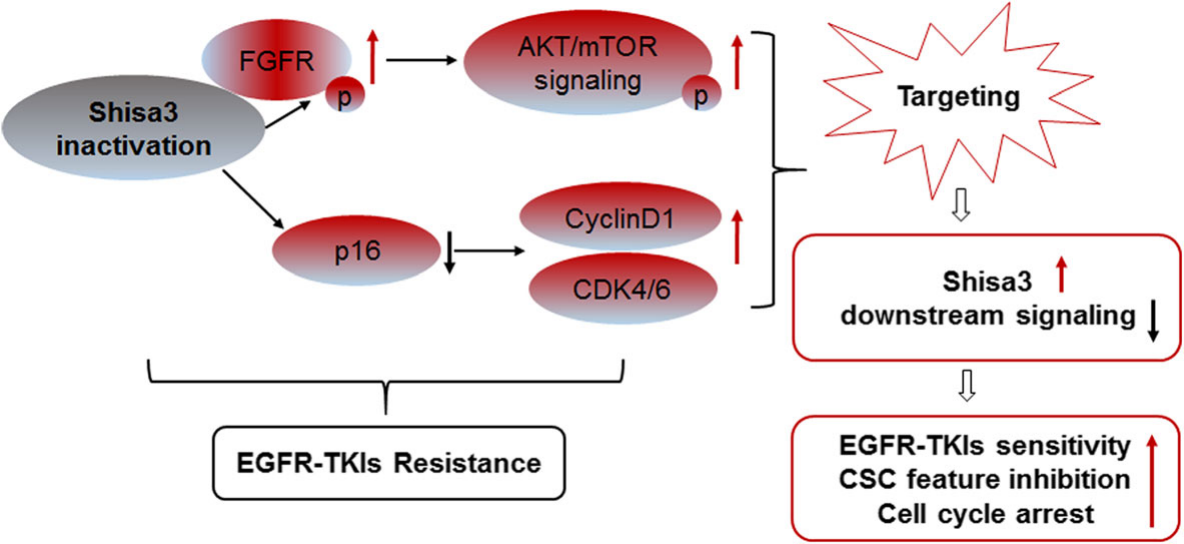
A schematic of shisa3-dependent FGFR/AKT/mTOR and cell cycle signaling that mediates EGFR-TKIs response and CSC phenotype in lung adenocarcinoma

Taken together, targeting shisa3-regulated signaling had an attenuated effect on EGFR-TKI-resistance that was associated with the depression of CSC properties (Fig. 7).

## Discussion

Although EGFR-TKIs benefit lung cancer patients with sensitive mutations, most patients eventually develop drug resistance and relapse. Previously, the role of CSCs in EGFR-TKI resistance had not been well clarified. Deep study of the molecular mechanisms of the regulating network in EGFR-TKI resistance is critical, as it may promote the development of novel therapeutic strategies to overcome a failed treatment. In the current study, we screened and verified that shisa3, as a suppressor, prevents EGFR-TKI resistance and suppresses the CSC phenotype in lung adenocarcinoma as follows: (1) Lung adenocarcinoma patients with high expression of shisa3 had a better response to EGFR-TKIs, indicating that shisa3 may be used to predict the efficacy of TKI therapy. (2) Shisa3 significantly suppressed self-renewal; expression levels of CSC-related factors; and migratory, invasive and tumorigenic capacities of CSC phenotypes, which can drive drug resistance in tumor cells. (3) Ectopic expression of shisa3 in vivo combined with gefitinib/osimertinib dramatically inhibited xenograft tumors from EGFR-TKI-resistant tumor cells. (4) Shisa3 altered the response of lung adenocarcinoma cells to EGFR-TKI treatment via FGFR/AKT/mTOR and cell cycle signaling. (5) TKIs combined with inhibitors of shisa3-regulated downstream signaling had enhanced function to restrain tumor growth in gefitinib/osimertinib-resistant xenografts, suggesting a potential therapeutic strategy to reverse EGFR-TKI resistance.

Increasing evidence has shown that acquired resistance to EGFR-TKIs is associated with an improved CSC phenotype [4, 23]. Herein, we found decreased shisa3 in gefitinib-resistant lung adenocarcinoma patients and EGFR-TKI-resistant PC9/ER cells. Consequently, shisa3 overexpression significantly controlled tumors derived from PC9/ER cells and CSC phenotypes. Shisa3 has been reported to accelerate the degradation of β-catenin in the Wnt signaling pathway, which regulates CSC maintenance in diverse types of cancer [24, 25]. Importantly, shisa3 combined with gefitinib and osimertinib inhibited tumor growth in PC9/ER xenografts, suggesting a potential role for this gene in reversing EGFR-TKI resistance.

Shisa3, located on chromosome 4p13, is a member of the shisa family, which mediates both WNT and FGF signaling by inhibiting the posttranslational maturation and cell surface trafficking of their receptors to cell surface [7]. Based on the above evidence showing the interaction of shisa with immature forms of FGFRs, we confirmed that ectopic shisa3 was immunoprecipitated with endogenous FGFR1 and FGFR3 in PC9/ER cells, indicating an interaction between shisa3 and FGFR1/3 that is involved in EGFR-TKI resistance. The FGF2-mediated FGFR/ERK pathway was previously considered to regulate CSCs, and inhibition of this signaling could delay tumor growth in esophageal squamous cell carcinoma [26]. Thus, it is reasonable to speculate that shisa3 decreased CSC characteristics by inhibiting the FGF-related axis. As we expected, shisa3 restored sensitivity to gefitinib/osimertinib and decreased CSC characteristics linked to FGFR1/3 activity.

Multiple mechanisms of EGFR-dependent and EGFR-independent resistance have been described. It is known that RTKs such as EGFR and FGFR mostly result in activation of downstream MAPK or PI3K/AKT/mTOR pathways. Drug resistance could be caused by the PI3K/AKT/mTOR signaling pathway, which is associated with CSC sustainability [27, 28]. Aberrant activation of the AKT pathway drives EGFR-TKI resistance that could be triggered by a variety of signaling [14]. FGFR induced by N-cadherin resulted in the phosphorylation of ERK and AKT to promote epithelial-mesenchymal transition (EMT) and CSC properties [29]. Our current findings suggest that shisa3 overcame EGFR-TKI resistance and CSC phenotypes by inactivating FGFR/AKT/mTOR and cell cycle signaling. We also found that EMT (down-regulation of E-cadherin, up-regulation of N-cadherin and vimentin) existed in the PC9/ER cells with resistance to gefitinib and osimertinib, compared to the parental PC9 cells. In addition, Met and HER2 amplification have been reported to drive EGFR-TKIs resistance [21]. In consistent with previous study, we also detected higher expression of Met in the PC9/ER cells. In summary, several mechanisms like decreased of shisa3, increased of Met and EMT activation lead to EGFR-TKIs resistance.

mTOR inhibition has been reported to suppress tumor growth in lung cancer cells and patient-derived xenograft (PDX) models [30]. In addition, an mTOR inhibitor that mediated cell cycle arrest displayed antitumor activity in preclinical evidence [31]. The inhibition of cyclin-dependent kinase 4/6 in vivo demonstrated a better erlotinib response to suppress TKI drug resistance in esophageal squamous cell carcinoma [32]. Currently, we found that TKIs combined with inhibition of the cell cycle and PI3K/AKT/mTOR signaling dramatically suppressed tumor growth in PC9/ER xenografts related to shisa3 activation.

## Conclusions

In summary, we demonstrated the crucial role of shisa3 in attenuating EGFR-TKI resistance, which is associated with CSC characteristics and the cell cycle. Our molecular studies indicated that shisa3 interacts with FGFR1/FGFR3 to decrease the activation of these two receptors and their downstream AKT/mTOR pathway, resulting in restoration of EGFR-TKI sensitivity and suppression of CSC properties in lung adenocarcinoma. Based on these essential findings, we suggest that shisa3 or cotargeting FGFR/AKT/mTOR and cell cycle signaling may be an effective therapeutic strategy for overcoming resistance to gefitinib and osimertinib.

## Supplementary information

**Additional file 1: Table S1.** QRT-PCR primer sequences. **Table S2.** EGFR mutations in lung adenocarcinoma patients. **Table S3.** EGFR mutations in lung adenocarcinoma cells. **Table S4.** mTOR signaling pathway enrichment in the PC9 cells with shisa3 knock-down. **Table S5.** Cell cycle signaling pathway enrichment in the PC9 cells with shisa3 knock-down. **Figure S1.** PC9/ER cells resistant to EGFR-TKIs induce CSC phenotype. **Figure S2.** Shisa3 decreases EGFR-TKI resistance and inhibits the CSC phenotype. **Figure S3.** EGFR-TKI inhibits tumor growth derived from PC9 cells in vivo.

## Data Availability

All data generated or analyzed during this study are included in this article. and its additional files.

## Abbreviations

CDS: Coding sequence
CSCs: Cancer stem cells
DFS: Disease-free survival
EGFR: Epidermal growth factor receptor
EMT: Epithelial-mesenchymal transition
FGFR: Fibroblast growth factor receptor
IC50: Half maximal inhibitory concentration
IHC: Immunohistochemistry
MAPK: Mitogen activated protein kinase
MET: Mesenchymal epithelial transition receptor
NCBI: The national center for biotechnology information
Nod-scid: Non-obese diabetic-severe combined immunodeficiency
NSCLC: Non-small cell lung cancer
OS: Overall survival
PI3K: Phosphoinositide-3-kinase
PR: Partial response
RTK: Receptor tyrosine kinase
SD: Stable disease
STAT3: Signal transducer and activator of transcription 3
TKI: Tyrosine kinase inhibitor
