# Supplementary material for: Shisa3 brakes resistance to EGFR-TKIs in lung adenocarcinoma by suppressing cancer stem cell properties
Source: J Exp Clin Cancer Res. 2019 Dec 4;38:481. doi: 10.1186/s13046-019-1486-3 (PMC6894286; doi:10.1186/s13046-019-1486-3)
Supplement: Supplementary file 1 — Additional file 1: Table S1. QRT-PCR primer sequences. Table S2. EGFR mutations in lung adenocarcinoma patients. Table S3. EGFR mutations in lung adenocarcinoma cells. Table S4. mTOR signaling pathway enrichment in the PC9 cells with shisa3 knock-down. Table S5. Cell cycle signaling pathway enrichment in the PC9 cells with shisa3 knock-down. Figure S1. PC9/ER cells resistant to EGFR-TKIs induce CSC phenotype. Figure S2. Shisa3 decreases EGFR-TKI resistance and inhibits the CSC phenotype. Figure S3. EGFR-TKI inhibits tumor growth derived from PC9 cells in vivo. [file 13046_2019_1486_MOESM1_ESM.docx]

Table S1. QRT-PCR primer sequences

| Gene Name | | Sense (5’ - 3’) | Anti-sense (5’ - 3’) | |
| --- | --- | --- | --- | --- |
| ABCG2 | | GGAGGCCTTGGGATACTTTGAA | GAGCTATAGAGGCCTGGGGATTAC | |
| ALDH1A1 | | CCCGTGGCGTACTATGGATG | CAGTGCAGGCCCTATCTTCC | |
| CD133 | | CACTACCAAGGACAAGGCGTTC | CAACGCCTCTTTGGTCTCCTTG | |
| CD44 | | CACACCCTCCCCTCATTCAC | CAGCTGTCCCTGTTGTCGAA | |
| E-cadherin | | GCCTCCTGAAAAGAGAGTGGAAG | TGGCAGTGTCTCTCCAAATCCG | |
| GAPDH | | GACCCCTTCATTGACCTCAAC | CTTCTCCATGGTGGTGAAGA | |
| HER-2 | | GGAAGTACACGATGCGGAGACT | ACCTTCCTCAGCTCCGTCTCTT | |
| MET | | TGCACAGTTGGTCCTGCCATGA | CAGCCATAGGACCGTATTTCGG | |
| Nanog | | TGCCTCACACGGAGACTGTC | TGCTATTCTTCGGCCAGTTG | |
| N-cadherin | | CCTCCAGAGTTTACTGCCATGAC | GTAGGATCTCCGCCACTGATTC | |
| Oct4 | | GACAACAATGAAAATCTTCAGGAGA | CTGGCGCCGGTTACAGAACCA | |
| PTEN | | TGAGTTCCCTCAGCCGTTACCT | GAGGTTTCCTCTGGTCCTGGTA | |
| Shisa3 | | CTCCATCTTCATTGCGTTCAT | TCGTGGCTGTGCTGGACT | |
| Sox2 | | ACATGAACGGCTGGAGCAAC | AGGAAGAGGTAACCACAGGG | |
| Vimentin | | AGGCAAAGCAGGAGTCCACTGA | ATCTGGCGTTCCAGGGACTCAT | |

Table S2. EGFR mutations in lung adenocarcinoma patients

| Patient | EGFR status | RECIST |
| --- | --- | --- |
| 1 | exon 19 deletion | PR |
| 2 | exon 19 deletion | PR |
| 3 | exon 21 mutation | PR |
| 4 | exon 21 mutation | SD |
| 5 | exon 21 mutation | SD |
| 6 | exon 21 mutation | SD |

PR: Partial Response; SD: Stable Disease

Table S3. EGFR mutations in lung adenocarcinoma cells

| Gene | Condons/exons | PC9 | PC9/GR | HCC827 | H1975 |
| --- | --- | --- | --- | --- | --- |
| EGFR | Exon 19 | Del 15 pb  (E746-A750) | Del 15 pb  (E746-A750) | Del 15 pb  (E746-A750) | Wild type |
|  | Exon 20 | Wild type | Wild type | Wild type | T790M |
|  | Exon 18,21 | Wild type | Wild type | Wild type | L858R |

Table S4. mTOR signaling pathway enrichment in the PC9 cells with shisa3 knock-down.

| **Gene Name** | **shShisa3 vs. shControl** | ***P* value** |
| --- | --- | --- |
| RPS6KA6 | 1.68 | <0.0001 |
| TBC1D7 | 1.57 | 0.00086 |
| PIK3R3 | 1.5 | 0.005129 |
| mTOR | 1.35 | 0.014786 |
| AKT1S1 | 1.35 | 0.000788 |
| RPS6KA2 | 1.34 | 0.003137 |
| eIF4E | 1.33 | 0.018194 |
| mLST8 | 1.31 | 0.00038 |
| PIK3R2 | 1.28 | 0.049126 |

Table S5. Cell cycle signaling pathway enrichment in the PC9 cells with shisa3 knock-down.

| **Gene Name** | **shShisa3 vs. shControl** | **P value** |
| --- | --- | --- |
| CDKN2B | 2.01 | 0.0465021 |
| CDC25A | 1.63 | 0.0210188 |
| CDK6 | 1.59 | 0.00675 |
| CCNE2 | 1.34 | 0.0215864 |
| CCNA2 | 1.32 | 0.0145777 |
| CCNB1 | 1.29 | 0.0125755 |
| CDK4 | 1.28 | 0.000682 |
| CDKN2A (P16) | 1.14 | 0.0419076 |
| CCND1 (cyclinD1) | 0.88 | 0.040293 |

**Figure legends**

**Figure S1**. PC9/ER cells resistant to EGFR-TKIs induce CSC phenotype. A. PC9 and PC9/ER cells were cultured in low-adherence conditions to form spheres in 96-well plates. Images of primary and secondary spheres are shown. Scale bars, 100 μm. **B**. The histogram shows the primary and secondary sphere formation efficiencies in PC9 and PC9/ER cells. **C**. The expression levels of CSC-related markers in PC9/ER cells compared to parental PC9 cells by qRT-PCR. **D**. Representative images of migratory and invasive cells of PC9 and PC9/ER cells. Scale bars, 100 μm. **E**. The migrated and invaded cells were quantiﬁed with ImageQuant. Data are presented as the means ± SDs of three independent assays. **F**. Tumorigenic images formed by transplanting 1×10^2^ and 1×10^3^ PC9/ER cells or PC9 cells into nod-scid mice (upper panel). Tumorigenic frequency was calculated by ELDA (<http://bioinf.wehi.edu.au/software/elda/>, lower panel). **G**. Higher expression levels of EGFR-TKI resistance-related genes were observed by qRT-PCR in PC9/ER cells than in parental PC9 cells. **H**. The expression levels of stem cell markers in PC9/ER cells compared to parental PC9 cells by qRT-PCR. **B**, **C,** **E, G** and **H**: n.s. p>0.05; *p<0.05; **p<0.001; ***p<0.0001.

**Figure S2**. Shisa3 decreases EGFR-TKI resistance and inhibits the CSC phenotype.

**A**. The upregulated mRNA and protein levels of Shisa3 were measured by qRT-PCR and Western blot in PC9/ER and H1975 cells stably transfected with Shisa3.

**B**, **C**. Assessment of proliferation of PC9/ER (**B**) and H1975 (**C**) cells transfected with Shisa3 or negative control by CCK-8 assay. **D**. Shisa3 expression was measured by qRT-PCR and Western blot in H1975 cells after doxycycline (2 μg/ml) treatment for 48 hours. **E**.CCK-8 assays were performed to assess the percentage of relative cell viability in H1975 cells exposed to gefitinib (10 μM, 20 μM) after doxycycline (2 μg/ml) treatment for 48 hours. **F**. The expression levels of shisa3 and stem cell markers in shisa3-overexpressing PC9/ER cells compared to the control cells by qRT-PCR. **G**. PC9/ER and shisa3-overexpressing PC9/ER cells were subjected to Transwell assays. Scale bars, 100 μm. **H**. The expression levels of shisa3 and stem cell markers in shisa3-downregulated PC9 cells compared to the control cells by qRT-PCR. **I**. Transwell assays demonstrated the number of migrated and invasive HCC827 cells transfected with the indicated shRNA. Scale bars, 100 μm. **A**, **B**, **C**, **E**, **F** and **H**: *p<0.05, **p<0.001, ***p<0.0001.

**Figure S3**. EGFR-TKI inhibits tumor growth derived from PC9 cells in vivo. **A**. Growth curves in the PC9 tumors with control (1% Tween 80 in PBS), osimertinib (5 mg/kg/d) or gefitinib (15 mg/kg/d) treatments at the indicated time points. Data are presented as the means ± SDs; n = 5. **B**. Representative xenograft images of PC9 tumors with the treatments. **C**. Histogram of tumor weights in PC9 tumors with control (1% Tween 80 in PBS), osimertinib (5 mg/kg/d) or gefitinib (15 mg/kg/d) treatments. **A**, **C**: **p<0.001, ***p<0.0001.
